# Interspecies Interactions between *Clostridium difficile* and *Candida albicans*

**DOI:** 10.1128/mSphere.00187-16

**Published:** 2016-11-09

**Authors:** Pim T. van Leeuwen, Jasper M. van der Peet, Floris J. Bikker, Michel A. Hoogenkamp, Ana M. Oliveira Paiva, Sarantos Kostidis, Oleg A. Mayboroda, Wiep Klaas Smits, Bastiaan P. Krom

**Affiliations:** a Department of Preventive Dentistry, Academic Centre for Dentistry Amsterdam (ACTA), Free University of Amsterdam and the University of Amsterdam, Amsterdam, The Netherlands; b Department of Oral Biochemistry, Academic Centre for Dentistry Amsterdam (ACTA), Free University of Amsterdam and the University of Amsterdam, Amsterdam, The Netherlands; c Department of Medical Microbiology, Leiden University Medical Center (LUMC), Leiden, The Netherlands; d Center for Proteomics and Metabolomics, Leiden University Medical Center (LUMC), Leiden, The Netherlands; University of Michigan

**Keywords:** *Candida albicans*, *Clostridium difficile*, interspecies interactions

## Abstract

The facultative anaerobic polymorphic fungus *Candida albicans* and the strictly anaerobic Gram-positive bacterium *Clostridium difficile* are two opportunistic pathogens residing in the human gut. While a few studies have focused on the prevalence of *C. albicans* in *C. difficile*-infected patients, the nature of the interactions between these two microbes has not been studied thus far. In the current study, both chemical and physical interactions between *C. albicans* and *C. difficile* were investigated. In the presence of *C. albicans*, *C. difficile* was able to grow under aerobic, normally toxic, conditions. This phenomenon was neither linked to adherence of bacteria to hyphae nor to biofilm formation by *C. albicans*. Conditioned medium of *C. difficile* inhibited hyphal growth of *C. albicans*, which is an important virulence factor of the fungus. In addition, it induced hypha-to-yeast conversion. *p*-Cresol, a fermentation product of tyrosine produced by *C. difficile*, also induced morphological effects and was identified as an active component of the conditioned medium. This study shows that in the presence of *C. albicans*, *C. difficile* can persist and grow under aerobic conditions. Furthermore, *p*-cresol, produced by *C. difficile*, is involved in inhibiting hypha formation of *C. albicans*, directly affecting the biofilm formation and virulence of *C. albicans*. This study is the first detailed characterization of the interactions between these two gut pathogens.

**IMPORTANCE** *Candida albicans* and *Clostridium difficile* are two opportunistic pathogens that reside in the human gut. A few studies have focused on the prevalence of *C. albicans* in *C. difficile*-infected patients, but none have shown the interaction(s) that these two organisms may or may not have with each other. In this study, we used a wide range of different techniques to better understand this interaction at a macroscopic and microscopic level. We found that in the presence of *C. albicans*, *C. difficile* can survive under ambient aerobic conditions, which would otherwise be toxic. We also found that *C. difficile* affects the hypha formation of *C. albicans*, most likely through the excretion of *p*-cresol. This ultimately leads to an inability of *C. albicans* to form a biofilm. Our study provides new insights into interactions between *C. albicans* and *C. difficile* and bears relevance to both fungal and bacterial disease.

## Abstract (precis)

*Candida albicans* and *Clostridium difficile* are two opportunistic pathogens that reside in the human gut. A few studies have focused on the prevalence of *C. albicans* in *C. difficile*-infected patients, but none have shown the interaction(s) that these two organisms may or may not have with each other. In this study, we used a wide range of different techniques to better understand this interaction at a macroscopic and microscopic level. We found that in the presence of *C. albicans*, *C. difficile* can survive under ambient aerobic conditions, which would otherwise be toxic. We also found that *C. difficile* affects the hypha formation of *C. albicans*, most likely through the excretion of *p*-cresol. This ultimately leads to an inability of *C. albicans* to form a biofilm. Our study provides new insights into interactions between *C. albicans* and *C. difficile* and bears relevance to both fungal and bacterial disease.

## INTRODUCTION

*Clostridium difficile* is a Gram-positive, obligate anaerobic, endospore-forming bacterium that is one of the most important causes of health care-associated infections (1, 2). Patients infected with *C. difficile* show symptoms that range from mild diarrhea to severe colitis—inflammation of the large intestine—that can lead to death (3). *C. difficile* infections (CDI) generally occur after use of broad-spectrum antibiotics that disrupt the normal gut microbiota. This dysbiosis permits *C. difficile* to colonize the large intestine, where the organism produces the toxins that are primarily responsible for the symptoms associated with CDI (4).

Since the early 2000s, there have been rising rates of CDI in Canada (5, 6), the United States (7), and Europe (8, 9). The current estimate is that 500,000 cases of CDI are diagnosed each year in the United States and 124,000 in Europe (1). Of these 500,000 cases, an estimated 4% of the patients never recover and eventually die (2, 10). The costs associated with CDI, an estimated $1 to $3 billion annually in the United States alone, represent a significant problem in health care settings (11, 12).

About 1 to 3% of hospitalized patients become infected with *C. difficile*, of which 25% will experience recurrent infections (13). These high relapse rates are partly due to the disruption of the healthy gut microbiota and the associated metabolome by antibiotics (14). Moreover, *C. difficile* is naturally resistant to several broad-spectrum antibiotics used in current medicine. The combined effect is that *C. difficile* can thrive while other bacteria in the intestinal flora suffer (15, 16). The risk of CDI declines once antibiotic treatment is completed and the gut microbiome can restore its original diversity and strength (2).

Like *C. difficile*, *Candida albicans* can be part of the gut microbiome of healthy individuals (17). *C. albicans* is a facultative anaerobic fungus that can grow in yeast, pseudohyphal, and hyphal morphologies and can persist in the gastrointestinal (GI) tract for prolonged periods of time (18). However, when the host’s immune system is compromised, *C. albicans* can cause a range of infections (19, 20). The virulence of *C. albicans* depends on its morphology; the filamentous morphology (called hypha) is invasive and poses a threat to the host, whereas the yeast and pseudohyphal morphologies do not (21–23).

The morphology of *C. albicans* is relevant to its interactions with other microorganisms. For instance, *Staphylococcus aureus* can invade the host by adhering to the hyphae of *C. albicans* (24). In addition, *C. albicans* can form heterogeneous biofilms with other microorganisms. These biofilms allow microorganisms to thrive under conditions that would normally be inhospitable (e.g., conferring vancomycin resistance to *S. aureus* [25–27]). Another bacterial species taking advantage of the microenvironment in the *C. albicans* biofilm is *Clostridium perfringens*, which can grow under normally toxic aerobic conditions when cocultured with *C. albicans* (26).

In addition to specific interactions between *Candida* and the host and *Candida* and the microbiome, *C. albicans* may be a key player of the gut mycobiota. The general role of mycobiota in the gastrointestinal tract is largely unexplored (28). Recent studies have illustrated a clear role for *C. albicans* in the postantibiotic recolonization of the cecum (29). This potentially decreases the chance of *C. difficile* relapse, as it shortens the duration of dysbiosis—the window of opportunity for *C. difficile*. Several recent studies have tried to define a possible correlation between *C. albicans* overgrowth and the presence of *C. difficile*. However, both positive (30–32) and negative correlations (33, 34) have been reported, and it remains uncertain how these two organisms interact.

In the current study, we investigated both the physical and chemical interactions between *C. albicans* and *C. difficile*. We show that *C. albicans* allows *C. difficile* to survive ambient oxygen levels in the absence of *C. albicans* biofilms and that hypha formation and subsequent biofilm formation by *C. albicans* are inhibited by *C. difficile*, most likely through the production of *p*-cresol.

## RESULTS

### Coculture with *C. albicans* allows *C. difficile* to grow and survive under aerobic conditions.

**FIG 1:**
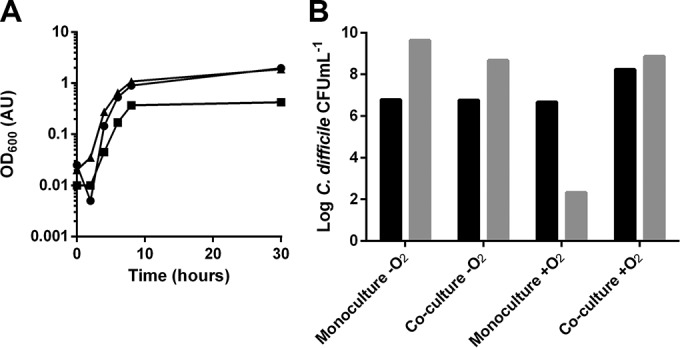
(A) OD_600_ values of monoculture of *C. difficile* (circles), monoculture of *C. albicans* (squares), and coculture of both organisms (triangles) under anaerobic conditions. All three conditions showed increasing OD_600_ values over time. The coculture of *C. difficile* with *C. albicans* and the monoculture of *C. difficile* reached similar OD_600_ values after 30 h. (B) Enumeration of *C. difficile* from monoculture and from coculture with *C. albicans* under anaerobic and aerobic conditions after 0 (black) and 24 (gray) h. Both monocultured and cocultured *C. difficile* grown under anaerobic conditions showed an increase in viable numbers. Under aerobic conditions, growth of monocultured *C. difficile* resulted in decreased viability, while *C. difficile* cocultured with *C. albicans* showed increased viability. Experiments were performed three times, and representative examples are shown.

While *C. albicans* is commonly cultured under aerobic conditions (35), it is able to grow anaerobically (36). *C. difficile*, however, is a strict anaerobic organism, and even small amounts of molecular oxygen are toxic (37). As the gut represents an anaerobic niche (38), the ability of *C. albicans*, *C. difficile*, and the combination of both organisms to grow under anaerobic conditions was determined. As expected, the optical density at 600 nm (OD_600_) of the *C. difficile* monoculture reached the stationary phase (OD_600_ of 1.97) after 30 h of growth (Fig. 1A), whereas the *C. albicans* monoculture showed limited growth (final OD_600_ of 0.43).

The coculture of *C. difficile* and *C. albicans* reached an OD_600_ value after 30 h that was comparable to that of the *C. difficile* monoculture. As the OD data do not prove whether *C. difficile* is responsible for this growth in coculture, additional plate counts were performed to determine bacterial growth. Anaerobic conditions supported the growth of *C. difficile* in monoculture, resulting in a 2-Log increase in viable counts (Fig. 1B). Under aerobic conditions, there was a 4-Log decrease in viability, consistent with the strict anaerobic nature of *C. difficile*. In contrast, coculturing *C. difficile* with *C. albicans* under aerobic conditions resulted in a 1-Log increase in the growth of *C. difficile*. These findings suggest that *C. difficile* is viable and able to replicate under aerobic conditions when cocultured with *C. albicans.*

### *C. difficile* does not adhere to hyphae of *C. albicans*.

The hyphae of *C. albicans* are attractive surfaces for bacteria to adhere to. Biofilm formation by the anaerobic bacterium *C. perfringens* on hyphae of *C. albicans* has been demonstrated previously (26). Because adherence is an important hallmark for heterogeneous biofilm formation, the adherence of *C. difficile* to *C. albicans* hyphae was evaluated using the Bioflux microfluidics platform.

The interaction of *C. difficile* with hyphae was limited under the conditions tested (see Fig. S1 in the supplemental material). Adhesion was mostly absent, but when bacteria adhered, they mostly did so at the tips of growing hyphae.

10.1128/mSphere.00187-16.1 Figure S1  Adhesion of *C. difficile* to hyphae of *C. albicans* is not readily apparent. When *C. difficile* was flowed over *C. albicans* hyphae in the Bioflux Z1000 setup under anaerobic conditions, adhesion rarely occurred (top row). However, when on occasion adhesion was observed, it was mostly located at the growing tips of hyphae (bottom row). The figure represents still images from a time-lapse capture. *C. difficile* was stained using *Bac*Light to enhance detection. The system was found to be (nearly) anaerobic within 15 min, based on measurements with the oxygen indicator resazurin (data not shown). Download Figure S1, TIF file, 2.6 MB. Copyright © 2016 van Leeuwen et al. 2016 van Leeuwen et al. This content is distributed under the terms of the Creative Commons Attribution 4.0 International license.

We conclude that, although *C. albicans* allows *C. difficile* to grow under normally toxic levels of oxygen, this is probably not mediated by aggregation or biofilm formation, in contrast to what has been suggested for other anaerobic bacteria.

### Coculture with *C. difficile* affects the morphology of *C. albicans*.

In the experiments described above, we established that *C. difficile* neither significantly adheres to hyphae nor stimulates biofilm formation of *C. albicans*. To investigate the presence of a chemical interaction between the two organisms, anaerobic growth in mono- and coculture was evaluated using microscopy.

**FIG 2:**
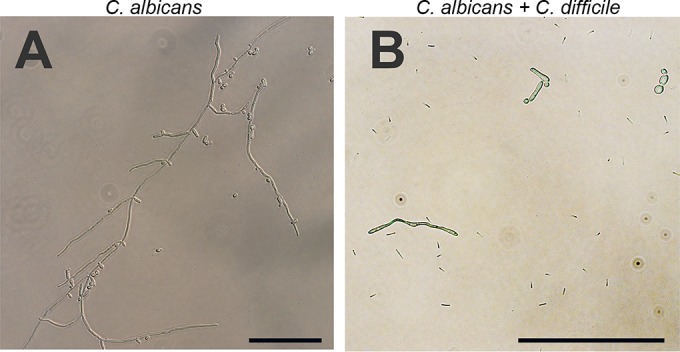
Morphology of *C. albicans* in anaerobic monoculture (A) and cocultured with *C. difficile* (B). In the monoculture, hyphae were clearly visible. Note that this is not one single hypha but multiple intertwined hyphae. In coculture with *C. difficile*, *C. albicans* was mostly observed in the yeast and pseudohyphal morphologies. All images have the same brightness and contrast adjustments. Scale bars indicate 100 µm; note that the scale for panel B is different to show the presence of *C. difficile* cells.

*C. albicans* is a polymorphic fungus, and the morphological switch is an important virulence factor (23). In anaerobic monoculture, *C. albicans* displayed predominantly hyphal growth, with the lengths of hyphae exceeding 100 µm after 4 h of growth (Fig. 2A). In coculture, this was different, with *C. albicans* growing mainly in yeast or pseudohyphal morphology (Fig. 2B).

### The morphological shift does not require the physical presence of *C. difficile*. 

**FIG 3:**
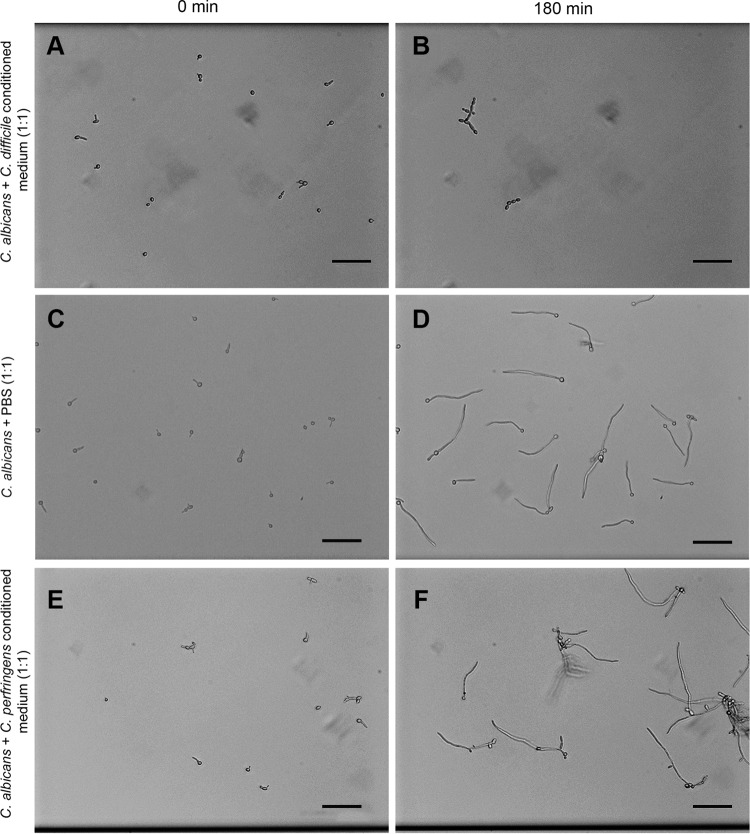
*C. albicans* grown in conditioned medium of *C. difficile* (A and B) and *C. perfringens* (E and F) at *t* = 0 min and *t* = 180 min. No hyphae are observed for *C. albicans* grown in *C. difficile*-conditioned medium, in contrast to substantial hypha formation by *C. albicans* growing in conditioned medium from *C. perfringens*. Note that lack of hyphae in *C. difficile*-conditioned medium caused some yeasts to wash out of the microchannel. All images have the same brightness and contrast adjustments. The scale bar indicates 50 µm.

Several bacterial species are known to secrete products that inhibit the yeast-to-hypha transition (39, 40). We therefore hypothesized that *C. difficile* produces a chemical signal that prevents and/or reverses hyphal growth of *C. albicans*. Such a chemical signal should persist in the absence of *C. difficile* cells. To investigate this, aerobically grown *C. albicans* was exposed to conditioned medium of *C. difficile*. We found that under such conditions, *C. albicans* grew almost exclusively in the yeast morphology (Fig. 3A and B), with few cells exhibiting a pseudohyphal morphology.

This effect cannot be attributed to nutrient depletion by dilution with the *C. difficile*-conditioned medium, as *C. albicans* exclusively showed hyphal growth when brain heart infusion (BHI) was diluted 1:1 with phosphate-buffered saline (PBS) (Fig. 3C and D). Moreover, the inhibition of hyphal growth seemed specific to *C. difficile*, as conditioned medium obtained from *C. perfringens* failed to inhibit the morphological switch (Fig. 3E and F).

### A stationary-phase signal produced by *C. difficile* can reverse hyphal growth of *C. albicans*.

Many bacterial products that affect morphological switching of *C. albicans* are produced during specific phases of growth (41, 42). We hypothesized that the *C. difficile*-dependent effects we describe above were also growth phase dependent. To test this hypothesis, the effects of conditioned medium derived from *C. difficile* cultures after 8, 24, and 48 h of incubation on *C. albicans* morphology were determined using real-time microscopy. This allowed a quantitative analysis of the fungal morphology over time.

**FIG 4:**
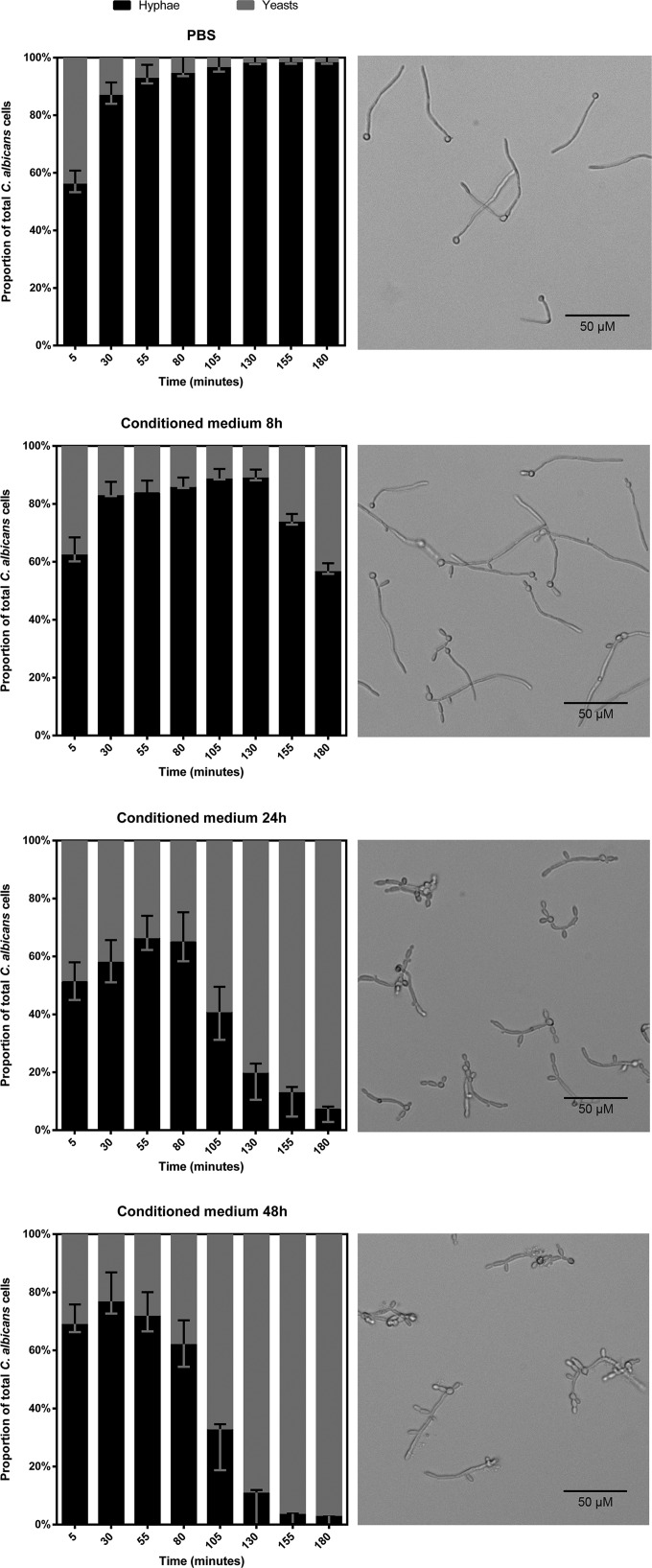
Proportions of *C. albicans* hyphae (black) and yeasts/pseudohyphae (gray) in different media. The images to the right of the graphs show the culture at 180 min. When grown in BHI, hyphae dominated the culture. The culture grown in 8-h-old *C. difficile*-conditioned medium yielded the same results until a morphological shift occurred at 130 min, after which the proportion of hyphae decreased in favor of the yeast and pseudohyphal morphologies. The shift occurred at earlier time points when 24-h and 48-h conditioned media were used, 55 min and 30 min, respectively. Moreover, the hypha-inhibiting effect is parallel to the age of the conditioned medium. The proportions of yeast and hyphal cells are significantly different between 24-h and 48-h conditioned medium and the control at the 130-min time point and thereafter (*P* < 0.05). Error bars indicate standard deviations. The scale bar indicates 50 µm.

In the time allowed for adherence to the microchannel, *C. albicans* initiated hypha formation; for this reason, the proportion of hyphae at 0 min is about 60% for all conditions. The control for nutrient depletion (BHI diluted 1:1 with PBS) showed increasing proportions of hyphae, up to 90%, compared to the proportions of yeasts and pseudohyphae during the time of the experiment (Fig. 4A). Upon the addition of conditioned medium from an 8-h-old culture of *C. difficile*, an increase from 60% to 80% hyphae in the first 2 h of exposure was noted, followed by a decline in the proportion of hyphae back to starting levels in the following hour. This demonstrates a reversal of hypha formation. The hypha-inhibiting effect was more pronounced when 24-h and 48-h *C. difficile*-conditioned medium was used, with less than 10% and 5% hyphal morphology, respectively, at the endpoint. Reversal of hypha formation also occurred at increasingly earlier time points in correlation with the age of the conditioned medium: at 130 min for the 8-h-old conditioned medium, 55 min for the 24-h-old conditioned medium, and 30 min for the 48-h-old conditioned medium (Fig. 4B to D).

We conclude that the chemical signal produced by *C. difficile* is most apparent in stationary growth phase.

### *p*-Cresol is involved in the inhibitory effect on hyphae formation of *C. albicans*.

We observed that the inhibitory effect on hyphae formation occurred for conditioned medium derived from *C. difficile* cultures and not for conditioned medium from *C. perfringens* cultures (Fig. 3). An important characteristic of *C. difficile* that sets it apart from other clostridia is its ability to produce *p*-cresol (4-methylphenol), and in contrast to other bacteria, *C. difficile* can grow in the presence of up to 0.1 to 0.2% (9.25 mM to 18.5 mM) of this compound (43–45). We fractionated the conditioned medium of both *C. difficile* and *C. perfringens* cultures using high-performance liquid chromatography (HPLC) and analyzed the activity of each fraction on *C. albicans* morphology.

**FIG 5:**
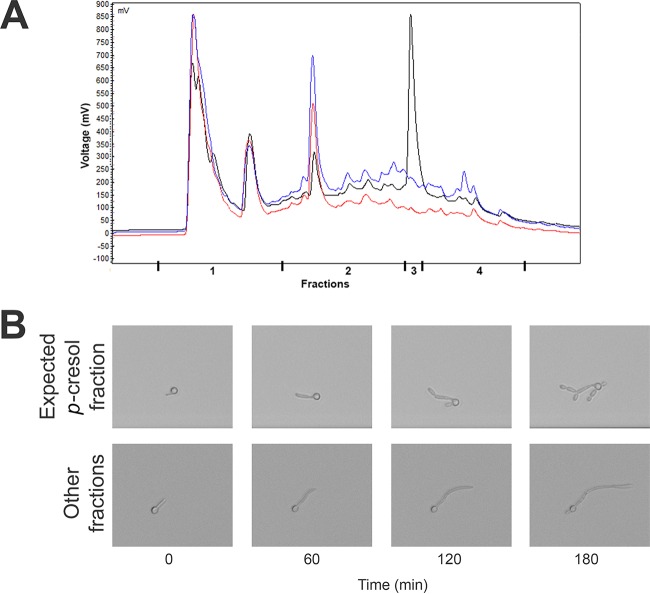
Fractions as they were collected (A) and effect on hyphae of *C. albicans* of the fraction expected to contain *p*-cresol compared to the effect of the other fractions (B). (A) The BHI spiked with 5% *p*-cresol (black) showed a clear peak at 13.1 min (fraction 3). Curves for conditioned media of *C. difficile* and *C. perfringens* are in blue and red, respectively. (B) The fraction that was expected to contain *p*-cresol (fraction 3) resulted in pseudohyphae. A clear time-dependent effect was observed when comparing the effect of fraction 3 to that of the other fractions. The magnification and time points of the sequences are identical.

To determine which fraction should contain the *p*-cresol, we used fresh BHI spiked with 5% *p*-cresol. We observed a sharp peak at 13.1 min in the elution profile (Fig. 5A). Analysis of the conditioned medium of *C. difficile* and *C. perfringens* after 24 h of growth showed minor peaks at this time point that could not clearly be attributed to *p*-cresol (Fig. 5A). However, when we compared the 13.1-min fraction (fraction 3) to all other fractions of the *C. difficile*-conditioned medium in our hypha formation assay, we observed that the fraction that was expected to contain *p*-cresol showed inhibition of hypha formation similar to that observed in previous assays with conditioned medium (Fig. 5B). It should be noted that the effect is an underestimate, due to dilution of the fraction with the HPLC eluate during elution from the HPLC.

**FIG 6:**
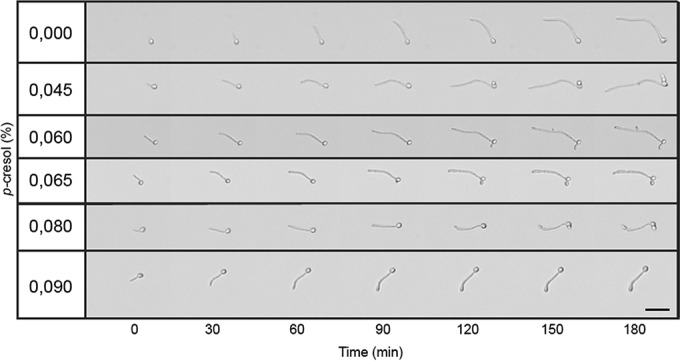
Effect of *p*-cresol on hypha formation of *C. albicans*. A concentration-dependent effect was observed, with the higher concentrations of *p*-cresol impairing hyphal elongation. Apparent swelling of the hyphal tip and yeast bud formation was observed. The scale bar indicates 25 µm.

To provide further evidence that *p*-cresol is capable of inhibiting hypha formation, we analyzed the effects of different concentrations of *p*-cresol on hypha formation over time. Because *p*-cresol is known to be toxic to bacteria in concentrations as low as 0.2% (46), we first determined whether *p*-cresol has a MIC for *C. albicans*. The observed MIC was 0.09% *p*-cresol (wt/vol), comparable to what has been observed for bacteria (47). The addition of *p*-cresol concentrations from 0.045% up to 0.09% in BHI resulted in inhibition of hypha formation in a concentration-dependent fashion (Fig. 6). Notably, *C. albicans* cultured in the higher concentrations of *p*-cresol showed strong swelling of the hyphal tips, consistent with a toxic effect.

**FIG 7:**
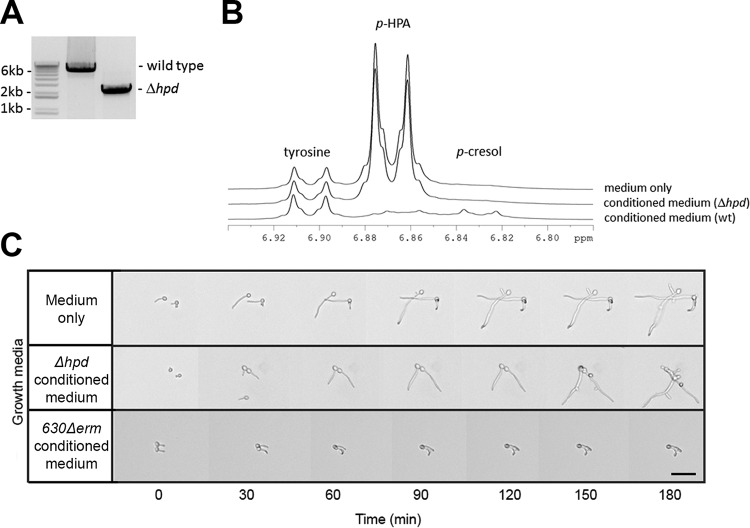
Effect of the Δ*hpd* mutant on hypha formation of *C. albicans*. (A) PCR analysis showing the expected 3.9-kb deletion of the *hpd* gene cluster. (B) The Δ*hpd* strain is unable to convert *p*-HPA to *p-*cresol. (C) Effects of 24-h-old conditioned medium of Δ*hpd* and wild-type *C. difficile* on hyphae of *C. albicans*. Hypha formation observed in conditioned medium of the Δ*hpd* mutant was similar to that in the control. In contrast, inhibition of hyphal growth was observed in the conditioned medium of wild-type *C. difficile*.

The formation of *p*-cresol from 4-hydroxyphenylacetic acid (*p*-HPA) requires the glycyl radical enzyme HpdB, a *p*-HPA decarboxylase, in *C. difficile* (48). The gene encoding this enzyme (*hpdB*/CD0153) is located in an operon that also encodes the proteins of unknown function HpdC (CD0154) and HpdA (CD0155), which are required for reconstitution of HpdB activity *in vitro* (48, 49). Insertional mutagenesis of either of these genes abolishes *p*-cresol production (50). To provide genetic evidence that *p*-cresol contributes to the observed effect on hypha formation, we generated an unmarked mutant of *C. difficile* in which the entire *hpd* operon was deleted by allelic exchange (51). PCR analysis showed the expected 3.9-kb deletion (Fig. 7A), and nuclear magnetic resonance (NMR) analysis confirmed that the mutant strain was unable to convert *p*-HPA to *p*-cresol (Fig. 7B). In contrast to conditioned medium from a wild-type *C. difficile* strain, conditioned medium from the *C. difficile* Δ*hpd* strain did not result in the inhibition of *C. albicans* hyphal elongation (Fig. 7C). Under all conditions, slight pseudohyphal growth was observed, possibly caused by the richness of the medium. Together, our data suggest that *p*-cresol, resulting from *p*-HPA decarboxylase activity, is important for the inhibitory effect of *C. difficile* on *C. albicans* hypha formation and elongation.

### **Wild-type** *C. difficile*, **but not the Δ*hpd* mutant, inhibits** *C. albicans*** biofilm formation.**

**FIG 8:**
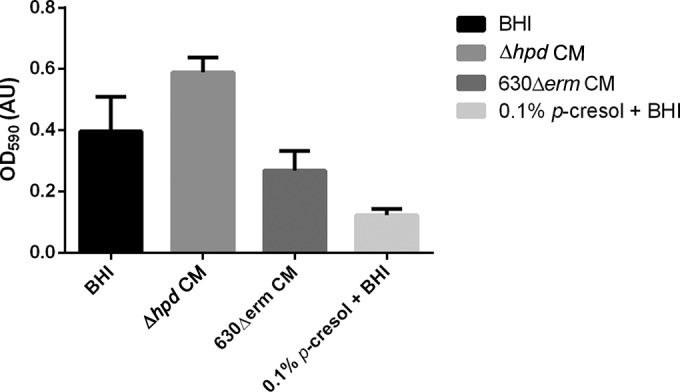
Effect of *p*-cresol on *C. albicans* biofilm formation. *C. albicans* biofilm formation is decreased in the presence of *C. difficile* 630Δ*erm*-conditioned medium (CM) (*P* < 0.05). *C. albicans* biofilm grown in the presence of 0.1% *p-*cresol is decreased more (*P* < 0.01). The *C. difficile* Δ*hpd*-conditioned medium showed increased biofilm formation. Data represent the results from 6 replicates, and error bars indicate standard deviations. We conclude that naturally produced and synthetic *p*-cresol can inhibit biofilm formation of *C. albicans*.

As hypha formation is crucial for the formation of *C. albicans* biofilms (52) and conditioned medium from *C. difficile* inhibits this process, we determined the effect of *C. difficile* on *C. albicans* biofilm formation. Biofilms of *C. albicans* were grown in fresh medium or 24-h-old conditioned medium of either wild-type or Δ*hpd C. difficile*. As a control, fresh medium supplemented with 0.1% *p*-cresol was included, which is expected to block biofilm formation by inhibition of *C. albicans* growth. Indeed, we observed a strong reduction in the biofilm assay (~fourfold, *P* < 0.01). We found that the conditioned medium from the wild-type *C. difficile* significantly reduced biofilm formation (~1.5-fold, *P* < 0.05) compared to that in fresh medium (Fig. 8). In contrast, conditioned medium from the *C. difficile* Δ*hpd* mutant stimulated biofilm formation ~1.5-fold compared to the biofilm formation in fresh medium. Since *C. albicans* biofilm formation is more pronounced in minimal medium than in rich medium, this could be related to nutrient depletion. Therefore, the actual difference in the biofilm-inhibitory effect between the Δ*hpd C. difficile* and the wild type is probably larger than comparison with the biofilm formation in fresh medium suggests.

## DISCUSSION

In this study, we investigated physical and chemical interactions between *C. difficile* and *C. albicans*. Two major discoveries are described: (i) *C. difficile* can survive ambient oxygen levels when cocultured with *C. albicans*, and (ii) *C. difficile* produces a secreted compound with inhibitory activity against two virulence factors of *C. albicans*—yeast-to-hypha transition and biofilm formation.

Multispecies biofilms are able to generate an anaerobic microenvironment that can sustain the growth of obligate anaerobic bacteria (26, 53, 54). This is exemplified by *C. albicans* and the anaerobic bacterium *C. perfringens* in a two-species system (26). To date, interactions of this nature between bacteria and *Candida* all rely on a physical interaction between the two species. Our study shows that this is not the case for *C. difficile*. Although *C. albicans* can sustain aerobic growth of *C. difficile*, we did not observe significant adherence of bacteria to hyphae of *C. albicans*. Moreover, *C. difficile* inhibited and reversed hypha formation, a key virulence factor and an essential process for *C. albicans* biofilm formation. What then underlies the positive effect of *C. albicans* on the aerobic survival and growth of *C. difficile*? We consider two, not mutually exclusive explanations. First, the facultative anaerobe *C. albicans* may reduce the oxygen tension through its metabolism (55), reducing it to levels that can sustain growth of *C. difficile*. It is important to note that culture methods strongly influence oxygen tension and the heterogeneity therein (56). Second, *C. albicans* may produce antioxidants. Indeed, *C. albicans* is known to produce tyrosol, which can act as an antioxidant, and it has recently been shown that the addition of strong antioxidants to culture medium allows the growth of anaerobic bacteria at ambient oxygen levels (57). In either case, there is an important implication of our finding. Vegetative *C. difficile* cells may be able to survive an oxygenated environment in the context of other species. In nature, most bacteria reside as polymicrobial communities in environments that are not necessarily anaerobic, such as the human oral and skin microbiome, and this greatly expands the ecological niches of *C. difficile* and, possibly, other obligate anaerobic bacteria.

The interactions between *C. difficile* and *C. albicans* bear relevance for both bacterial and fungal disease. Several studies have evaluated the relation between CDI and *Candida* colonization and/or disease, and both positive and negative associations have been reported. Patients treated for CDI were found to be able to acquire *Candida*, while *Candida* levels in precolonized patients showed a reduction of *C. albicans* during treatment (34). CDI patients were less likely to have *C. albicans* overgrowth (33), but *C. albicans* bloodstream infections were reported after a CDI episode (31). Finally, CDI-positive patients were reported to have higher *C. albicans* colonization rates (30). The interpretation of these findings is difficult, as the reports are largely observational and diverse in patient status (e.g., antibiotic treatment). As both *C. albicans* and *C. difficile* are opportunistic pathogens, differences in treatment could result in an environment that favors one pathogen over the other.

In this study, we found that *C. albicans* allowed *C. difficile* to grow at ambient oxygen levels. It is believed that there is an oxygen gradient from the proximal (stomach) to distal (rectum) gastrointestinal tract (38) that can affect bacterial virulence and host responses (58) and plays a role in intestinal dysbiosis (59). It is therefore conceivable that *C. difficile* in hosts colonized by *Candida* can, under suitable conditions (such as high levels of primary bile acids) (60), colonize a greater niche than the colon alone.

The presence of *C. difficile* was linked to an absence of *C. albicans* hyphae. This suggests that invasive *Candida* infections, originating in the GI tract and potentially leading to candidemia, are less likely to occur in CDI patients. As *C. difficile* did not inhibit proliferation of the yeast form of *C. albicans*, superficial candidiasis could still be possible. Inhibition of *Candida* hypha formation by *C. difficile* may also be relevant for recurrent CDI. A positive effect of *C. albicans* on the regeneration of the intestinal flora after antibiotic-induced dysbiosis was recently shown (29), and reestablishment of a diverse gut microbiome is accompanied by a decreasing probability for the development of CDI (2, 10). By inhibiting hypha formation, *C. difficile* can prolong the window of opportunity for the development of CDI as it competes directly with *C. albicans* itself or by competing with bacteria in a heterogeneous *C. albicans*-containing biofilm.

The results from this study suggest an involvement of *p*-cresol, produced by *C. difficile*, in the inhibitory effect on *C. albicans* hypha formation. Cell-free conditioned medium from wild-type *C. difficile*, but not from an isogenic Δ*hpd* strain, inhibited hypha formation (Fig. 2 to 5, 7, and 8) and reduced biofilm formation of *C. albicans* (Fig. 8). Fractionation of the conditioned medium showed that the *p*-cresol-containing fraction inhibited hypha formation, whereas the other fractions did not. Conditioned medium from *C. perfringens*, which does not produce *p*-cresol (45, 61), failed to inhibit hyphal formation and biofilm formation. Pure *p*-cresol was capable of stopping hyphal growth and induced swelling of the hyphal tips (Fig. 6). However, we were unable, to detect *p*-cresol in conditioned BHI medium (Fig. 5A), consistent with previous findings (50). To validate our *C. difficile* Δ*hpd* strain (Fig. 7B), medium supplemented with the biosynthetic precursor *p*-HPA was used, as previously described (50). Both the fractionation of the conditioned medium and the NMR analysis of the *p*-cresol content of the medium required extensive processing of the sample. As *p*-cresol is a volatile compound, this could explain our failure to detect it in these experiments. Consistent with this, we note that the complete conversion of *p*-HPA by the wild-type *C. difficile* is accompanied by very minor peaks corresponding to *p*-cresol (Fig. 7B). Though all our experiments support the involvement of *p*-cresol in the inhibition of hypha formation, we cannot exclude the possibility that *C. difficile* produces other compounds of metabolic products that also affect *C. albicans* morphology.

Previously, *p*-cresol was identified as an antibacterial, and it has been exploited to facilitate the isolation of *C. difficile* (45, 46). The MIC of *p*-cresol for *C. albicans* observed in this study (0.09% wt/vol) is comparable to those for bacteria. To our knowledge, this is the first time that an antifungal effect of *p*-cresol has been reported. We cannot exclude the possibility that part of the effect of *p*-cresol on the morphology of *C. albicans* is related to toxicity. Other signaling compounds (e.g., farnesol [reviewed in reference 62]) and antifungals (e.g., fluconazole [63]) are also toxic at higher levels. Hyphal inhibitors are already being used to treat candidiasis (64, 65), and our finding may lead to enhanced treatment options for *C. albicans* infections. The mode of action of *p*-cresol toward *C. albicans* is unknown. However, the effects on *Candida* of two compounds that share structural similarities to *p*-cresol, thymol and carvacrol, have been reported (66). Both compounds cause oxidative stress, damage the antioxidant defense system, and lead to membrane deterioration. It is tempting to speculate that a similar mechanism contributes to the action of *p*-cresol. Notably, several other phenolic compounds have been reported to positively or negatively affect *Candida* viability, morphology, and biofilm formation; these include but are not limited to tyrosol (67, 68), *p*-coumaric acid (69), ferulic acid (70), caffeic acid (71), boric acid (72), and eugenol (73). These observations, together with the identification of more-complex inhibitors (74, 75), may lead to the identification of general structural principles of compounds that govern *Candida* proliferation and the yeast-to-hypha transition.

## MATERIALS AND METHODS

### Strains and growth conditions.

The strains used in this study were *C. difficile* 630Δ*erm* (76, 77) and an isogenic *hpd* mutant (this study), *C. perfringens* ATCC 13124 (78), and *C. albicans* SC5314 (79). Frozen stocks were prepared by adding sterile glycerol (final concentration, 10% [vol/vol]) to a fresh culture and storing at −80°C.

All medium components were purchased at BD unless stated otherwise. Both clostridiae were routinely subcultured on brain heart infusion (BHI) containing 1.5% [wt/vol] Bacto agar at 37°C under anaerobic conditions (10% H_2_, 10% CO_2_ in N_2_). *C. albicans* was cultured on yeast-peptone-dextrose (YPD) agar (1% [wt/vol] Bacto yeast extract, 2% [wt/vol] Bacto peptone, 2% [wt/vol] dextrose [Merck], 1.5% [wt/vol] Bacto agar) at 30°C under aerobic conditions.

Prior to each experiment, fresh planktonic cultures were prepared by inoculating a single colony of each strain to either BHI or YPD (for both media, the agar was omitted). The clostridia were anaerobically incubated at 37°C for 72 h under agitation. *C. albicans* was incubated aerobically at 30°C for 16 h under agitation.

### Construction of the *C. difficile* Δ*hpd* strain.

The up- and downstream regions of the *hpd* operon that are required for *p*-cresol synthesis (48, 50) were cloned into the pMTL-SC7215 vector (51) using Gibson assembly (80). Primer design was carried out using the NEBuilder tool version 1.10.7 (New England Biolabs), with a minimum overlap length of 30 bp, as seen in Table 1. All PCRs were carried out on chromosomal DNA of *C. difficile* 630Δ*erm* (76, 77) using Q5 polymerase (New England Biolabs). The region upstream (~950 bp) and the coding region for the first 3 amino acids of *hpdB* were amplified using primers oWKS-1545 and oWKS-1546. The coding region for the last 3 amino acids of *hpdA* and the downstream region (~950 bp) were amplified using oWKS-1547 and oWKS-1548. The vector was amplified from pMTL-SC7215 using primers oWKS-1537 and oWKS-1538. One hundred nanograms of vector DNA was assembled with a threefold excess of the two other PCR fragments using Gibson Assembly Master Mix (New England Biolabs) for 30 min at 50°C and transformed into *Escherichia coli* DH5α. Transformants were screened by colony PCR using primers oWKS-1539 and oWKS-1540. Plasmids with the correct insert size were verified by Sanger sequencing, yielding pWKS1811. Purified pWKS1811 was transformed into *E. coli* CA434 (81), which was subsequently used as a donor to introduce the plasmid into *C. difficile* 630Δ*erm* by conjugation. Transconjugants were selected on BHI agar supplemented with yeast extract (5 g/liter; Sigma), 15 µg/ml thiamphenicol, and *C. difficile*-selective supplement (CDSS; Oxoid) (BYTC plates) for 6 days. Several colonies were patched to fresh plates and checked for single-crossover integration of the pWKS1811 plasmid using PCR. Such a clone was subsequently plated onto BHI agar with CDSS but without thiamphenicol and incubated for 96 h to allow the second crossover event to occur. Colonies from this plate were harvested into 500 µl PBS, serially diluted, and plated onto minimal agar containing 50 µg/ml of 5-fluorocytosine (Sigma) (CDMM-5FC plates) (51). Colonies were screened for loss of the plasmid by patching on fresh BYTC and CDMM-5FC plates. Colonies that had lost the plasmid, as evidenced by thiamphenicol sensitivity, were grown in BHI broth supplemented with 5 g/liter yeast extract, and DNA was isolated using the DNeasy blood and tissue kit (Qiagen). The presence of the chromosomal deletion was verified by PCR using primers oWKS-1549 and oWKS-1550 and Sanger sequencing of the PCR product using primers oWKS-1545 and oWKS-1548. This yielded an unmarked *C. difficile* Δ*hpd* strain (AP58) with the entire up- and downstream region of the operon intact but with the *hpdBCA* coding regions effectively removed.

**TABLE 1:** Oligonucleotides used in this study

Name	Sequence (5′→3′)	Description^a^
oWKS-1537	TAGGGTAACAAAAAACACCG	RF311; reverse primer for amplification of vector (85)
oWKS-1538	CCTTTTTGATAATCTCATGACC	RF312; forward primer for amplification of vector (85)
oWKS-1539	GGATTTCACATTTGCCGTTTTGTAAAC	RF21; forward primer for screening inserts in vector (85)
oWKS-1540	GATCTTTTCTACGGGGTCTGAC	RF22; for screening inserts in vector (85)
oWKS-1545	CGTAGAAATACGGTGTTTTTTGTTACCCTAATCTGGAGGTCATACTCAC	Forward primer for upstream region of *hpd* operon
oWKS-1546	TTAATTTTAGAAAGCTTGACTCATTTCTTCCCC	Reverse primer for upstream region of *hpd* operon
oWKS-1547	GAAGAAATGAGTCAAGCTTTCTAAAATTAAATACAAGTTTTAATTAAAAAAG	Forward primer for downstream region of *hpd* operon
oWKS-1548	GGGATTTTGGTCATGAGATTATCAAAAAGGTAACAGATGGAACAATCATTATAAAATAAATATTTTTAC	Reverse primer for downstream region of *hpd* operon
oWKS-1549	TGGTGGTGTAGTTCCAGAAG	Forward primer in the *gcp* gene, upstream from the *hpd* operon
oWKS-1550	GAAGTCCACTTACAGGCATACC	Reverse primer in *cd0156*, downstream from the *hpd* operon

a RF311, RF312, RF21, and RF22 are the primer designations used in reference 85.

### Coculturing experiments.

The effect of *C. albicans* on *C. difficile* growth was determined by diluting fresh cultures of both strains to an optical density at 600 nm (OD_600_) of 0.01 in a total volume of 10 ml and incubating for 30 h under anaerobic conditions at 37°C in test tubes. Growth was assessed by determining the OD_600_ at 0, 2, 4, 6, 8, and 30 h after inoculation.

The growth of the individual species in both monoculture and coculture was assessed at all time points by performing serial dilution and spiral plating the samples on either YPD agar or BHI agar supplemented with 5% (vol/vol) defibrinated sheep blood (Biotrading Benelux, Mijdrecht, the Netherlands). Both types of plates were incubated under both aerobic and anaerobic conditions for 24 h, as aerobic conditions do not support the growth on plates of *C. difficile* and anaerobic conditions do not support the growth of *C. albicans* on BHI agar within 24 h.

### Adhesion assay.

Adhesion of bacteria was analyzed using the Bioflux Z1000 platform (Fluxion Biosciences, Inc., South San Francisco, CA, USA) as described previously (82). The Bioflux was operated using the anaerobic air mixture, which resulted in (near) anaerobiosis of the channel within 15 min (unpublished observations). Briefly, the microscope stage was heated to 37°C and all solutions used were warmed to 37°C prior to use. PBS was flowed through the channels at 1 dyn/cm^2^ until the channels were free of air. Subsequently, 10% fetal bovine serum (product number F7524; Sigma) in PBS was flowed through the channels of a Fluxion 48-well plate for 30 min to coat the channels. *C. albicans* in yeast nitrogen base containing 0.5% glucose (YNB) at an OD_600_ of 0.1 was introduced into the channels from the output well. Subsequently, the flow was stopped and *C. albicans* was allowed to adhere for 15 to 30 min. Then, YNB was flowed through the channels at 0.5 dyn/cm^2^ to allow for hypha formation for 2.5 h. *C. difficile* from a liquid preculture was harvested by centrifugation at 10,000 × *g* for 1 min and resuspended in BHI to an OD_600_ of 0.2. These cells were then stained with LIVE/DEAD BacLight bacterial viability stain (Invitrogen) and passed over the hyphae at 0.5 dyn/cm^2^. Adhesion was visualized every minute for a total of 25 min using bright-field and fluorescent filter sets (60× objective; for fluorescein isothiocyanate [FITC], excitation wavelengths were 475/40 nm and band-pass [BP] wavelengths were 530/50 nm, and for red fluorescent dye, excitation wavelengths were 545/25 nm and BP wavelengths were 605/70 nm). Images were analyzed using ImageJ version 1.49 (83).

### Preparation of conditioned medium.

To prepare conditioned medium, fresh cultures of each strain were grown as described above. Bacteria were removed by centrifugation (5,000 × *g* for 10 min), and the supernatant was subsequently filter sterilized (0.2 µm) and stored at −20°C until further use.

### Hypha formation assay.

To assess the influence of the conditioned medium on hypha formation, the BioFlux microfluidics platform (Fluxion) was used. Briefly, *C. albicans* yeast cells (OD_600_ of 0.1) were allowed to adhere to the surface as described above for the adhesion assay. After 30 min, conditioned medium was continuously introduced into the channel at 0.5 dyn/cm^2^. Hypha formation was visualized using automated bright-field microscopy (Carl Zeiss Observer Z1) at 3 different locations per channel every 5 min for 3 h. The images were analyzed using ImageJ version 1.49 (83).

### Fractionation of conditioned medium.

To investigate which secreted component was responsible for the effect on hypha formation and adherence, conditioned medium was fractionated. Fresh BHI spiked with 5% *p*-cresol and *C. difficile*-conditioned BHI were run on a C_4_ column (Vydac 214TP C_4_; Grace Davison Discovery Sciences) using a reverse-phase HPLC system (AS-1555, PU-980, LG-980-02, and DG-980-50; Jasco) equilibrated in 0.1% trifluoroacetic acid. Elution was performed with a linear gradient of 30 to 45% acetonitrile containing 0.1% trifluoroacetic acid in 30.5 min at a flow rate of 4 ml/min. The absorbance of the effluent was monitored at 214 nm, and fractions were collected separately using a fraction collector. The fractions were lyophilized in a rotational vacuum concentrator (RVC 2-25 plus; Martin Christ) and stored at −20°C until further use.

### *p*-Cresol MIC assay.

To assess the MIC of *p*-cresol toward *C. albicans*, a planktonic culture was prepared as described above. The range of concentrations of *p*-cresol (0.1% to 0% with 0.01% [wt/vol] increments in BHI) were prepared in final volumes of 990 µl. Subsequently, 10 µl *C. albicans* culture was added to each *p*-cresol dilution to a final OD_600_ of ≈0.05. A 200-µl amount of each dilution was added in triplicate to the wells of a microtiter plate (Greiner), and the plates incubated for 16 h at 30°C under aerobic conditions. Prior to determining the final OD_600_ and the MIC, all suspensions were resuspended by pipetting to homogenize the well contents.

### NMR validation of the *C. difficile* Δ*hpd* strain.

All chemicals used for sample preparation were purchased from Sigma-Aldrich (Germany), except for sodium trimethylsilylpropionate-d4 (TMSP-2,2,3,3-D4 [TMSP]), which was purchased from Cambridge Isotope Laboratories, Inc. (United Kingdom). Frozen conditioned medium samples were allowed to thaw at 4°C. A 400-µl amount of each sample was mixed with 800 µl of ice-cold methanol in an Eppendorf tube and immediately placed at −30°C for 10 min to initiate protein precipitation. Subsequently, the mixtures were centrifuged at 16,000 × *g* at 4°C for 20 min, and the supernatants were collected and dried under a nitrogen gas stream. The dried material was reconstituted with 0.25 ml of phosphate buffer solution in D_2_O (150 mM K_2_HPO_4_, pH 7.4), including 0.4 mM of TMSP as the chemical shift reference standard for proton NMR, and transferred to 3-mm NMR tubes. All NMR data were recorded on a 14.1 T (600 MHz, 1 H) Bruker Avance II NMR spectrometer (Bruker Biospin, GmbH, Germany) equipped with a 5-mm TCI cryoprobe. One dimensional (1-D) proton NMR experiments were measured at 27°C, using the 1-D nuclear Overhauser effect spectroscopy (NOESY) with presaturation and spoil gradients (noesygppr1d) pulse sequence as implemented in the Topspin 3.0 library (Bruker Biospin GmbH, Germany), with presaturation for water signal suppression. Per sample, 65,536 data points were collected with 512 scans for a total of 59 min of acquisition time. Spectral data were Fourier transformed, phased, baseline corrected, and referenced to the TMSP peak at 0.00 ppm. The peaks of *p*-cresol, *p*-hydroxyphenylacetate (*p*-HPA), and l-tyrosine were annotated using the reference spectra of the Bruker Biorefcode database and by 2-dimensional (2-D) NMR spectroscopy (data not shown).

### Crystal violet assay.

Biofilms were allowed to form in a microtiter plate as previously described (84). Four different media were introduced to the wells before incubation: BHI, *C. difficile* Δ*hpd*-conditioned medium (Δ*hpd* CM), *C. difficile* 630Δ*erm*-conditioned medium (630Δ*erm* CM), and BHI spiked with 0.1% *p-*cresol*.* After incubation, wells were thoroughly washed with PBS, followed by the addition of 0.2% (wt/vol) crystal violet in demineralized water. After 10 min of incubation, wells were washed with PBS to remove excess crystal violet. To quantify biofilm formation, crystal violet was extracted from the cells using isopropanol with 1 N HCl, and the absorption was determined at 590 nm.

### Statistical analyses.

The significance of the data was analyzed using Student’s *t* test. Differences were deemed significant if the *P* value was <0.05.

## References

[1] Smits WK, Lyras D, Lacy BD, Wilcox MH, Kuijper EJ 2016 *Clostridium difficile* infection. Nat Rev Dis Primers 2:16020. doi:10.1038/nrdp.2016.20. 27158839 PMC5453186

[2] Abt MC, McKenney PT, Pamer EG 2016 *Clostridium difficile* colitis: pathogenesis and host defence. Nat Rev Microbiol 14:609–620. doi:10.1038/nrmicro.2016.108. 27573580 PMC5109054

[3] Shen A 2012 *Clostridium difficile* toxins: mediators of inflammation. J Innate Immun 4:149–158. doi:10.1159/000332946. 22237401 PMC3388264

[4] Seekatz AM, Rao K, Santhosh K, Young VB 2016 Dynamics of the fecal microbiome in patients with recurrent and nonrecurrent *Clostridium difficile* infection. Genome Med 8:1. doi:10.1186/s13073-016-0298-8. 27121861 PMC4847246

[5] Pépin J, Valiquette L, Alary ME, Villemure P, Pelletier A, Forget K, Pépin K, Chouinard D 2004 *Clostridium difficile*-associated diarrhea in a region of Quebec from 1991 to 2003: a changing pattern of disease severity. CMAJ 171:466–472. doi:10.1503/cmaj.1041104. 15337727 PMC514643

[6] Labbé AC, Poirier L, Maccannell D, Louie T, Savoie M, Béliveau C, Laverdière M, Pépin J 2008 *Clostridium difficile* infections in a Canadian tertiary care hospital before and during a regional epidemic associated with the BI/NAP1/027 strain. Antimicrob Agents Chemother 52:3180–3187. doi:10.1128/AAC.00146-08. 18573937 PMC2533448

[7] Dallal RM, Harbrecht BG, Boujoukas AJ, Sirio CA, Farkas LM, Lee KK, Simmons RL 2002 Fulminant *Clostridium difficile*: an underappreciated and increasing cause of death and complications. Ann Surg 235:363–372. doi:10.1097/00000658-200203000-00008. 11882758 PMC1422442

[8] Kuijper EJ, Barbut F, Brazier JS, Kleinkauf N, Eckmanns T, Lambert ML, Drudy D, Fitzpatrick F, Wiuff C, Brown DJ, Coia JE, Pituch H, Reichert P, Even J, Mossong J, Widmer AF, Olsen KE, Allerberger F, Notermans DW, Delmee M, Coignard B, Wilcox M, Patel B, Frei R, Nagy E, Bouza E, Marin M, Akerlund T, Virolainen-Julkunen A, Lyytikainen O, Kotila S, Ingebretsen A, Smyth B, Rooney P, Poxton IR, Monnet DL 2008 Update of *Clostridium difficile* infection due to PCR ribotype 027 in Europe, 2008. Euro Surveill 13:pii=18942 http://www.eurosurveillance.org/ViewArticle.aspx?ArticleId=18942. 18761903

[9] Kuijper EJ, Coignard B, Tüll P, ESCMID Study Group for Clostridium difficile, EU Member States, European Centre for Disease Prevention and Control 2006 Emergence of *Clostridium difficile*-associated disease in North America and Europe. Clin Microbiol Infect 12(Suppl 6):2–18. doi:10.1111/j.1469-0691.2006.01580.x. 16965399

[10] Rupnik M, Wilcox MH, Gerding DN 2009 *Clostridium difficile* infection: new developments in epidemiology and pathogenesis. Nat Rev Microbiol 7:526–536. doi:10.1038/nrmicro2164. 19528959

[11] McGlone SM, Bailey RR, Zimmer SM, Popovich MJ, Tian Y, Ufberg P, Muder RR, Lee BY 2012 The economic burden of *Clostridium difficile*. Clin Microbiol Infect 18:282–289. doi:10.1111/j.1469-0691.2011.03571.x. 21668576 PMC3763211

[12] O’Brien JA, Lahue BJ, Caro JJ, Davidson DM 2007 The emerging infectious challenge of *Clostridium difficile*-associated disease in Massachusetts hospitals: clinical and economic consequences. Infect Control Hosp Epidemiol 28:1219–1227. doi:10.1086/522676. 17926270

[13] Carroll KC, Bartlett JG 2011 Biology of *Clostridium difficile*: implications for epidemiology and diagnosis. Annu Rev Microbiol 65:501–521. doi:10.1146/annurev-micro-090110-102824. 21682645

[14] Johanesen PA, Mackin KE, Hutton ML, Awad MM, Larcombe S, Amy JM, Lyras D 2015 Disruption of the gut microbiome: *Clostridium difficile* infection and the threat of antibiotic resistance. Genes 6:1347–1360. doi:10.3390/genes6041347. 26703737 PMC4690045

[15] Gerding DN, Johnson S 2010 Management of *Clostridium difficile* infection: thinking inside and outside the box. Clin Infect Dis 51:1306–1313. doi:10.1086/657116. 20979491

[16] Spigaglia P, Barbanti F, Mastrantonio P, European Study Group on Clostridium difficile (ESGCD) 2011 Multidrug resistance in European *Clostridium difficile* clinical isolates. J Antimicrob Chemother 66:2227–2234. doi:10.1093/jac/dkr292. 21771851

[17] Kennedy MJ, Volz PA, Edwards CA, Yancey RJ 1987 Mechanisms of association of *Candida albicans* with intestinal mucosa. J Med Microbiol 24:333–341. doi:10.1099/00222615-24-4-333. 3320372

[18] Ruiz-Sánchez D, Calderón-Romero L, Sánchez-Vega JT, Tay J 2002 Intestinal candidiasis. A clinical report and comments about this opportunistic pathology. Mycopathologia 156:9–11. doi:10.1023/A:1021326713470. 12715941

[19] Metwalli KH, Khan SA, Krom BP, Jabra-Rizk MA 2013 *Streptococcus mutans*, *Candida albicans*, and the human mouth: a sticky situation. PLoS Pathog 9:e1003616. doi:10.1371/journal.ppat.1003616. 24146611 PMC3798555

[20] Brown GD, Denning DW, Gow NA, Levitz SM, Netea MG, White TC 2012 Hidden killers: human fungal infections. Sci Transl Med 4:165rv113. doi:10.1126/scitranslmed.3004404. 23253612

[21] Lo HJ, Köhler JR, DiDomenico B, Loebenberg D, Cacciapuoti A, Fink GR 1997 Nonfilamentous *C. albicans* mutants are avirulent. Cell 90:939–949. doi:10.1016/S0092-8674(00)80358-X. 9298905

[22] Murad AM, Leng P, Straffon M, Wishart J, Macaskill S, MacCallum D, Schnell N, Talibi D, Marechal D, Tekaia F, d’Enfert C, Gaillardin C, Odds FC, Brown AJ 2001 NRG1 represses yeast-hypha morphogenesis and hypha-specific gene expression in *Candida albicans*. EMBO J 20:4742–4752. doi:10.1093/emboj/20.17.4742. 11532938 PMC125592

[23] Mayer FL, Wilson D, Hube B 2013 *Candida albicans* pathogenicity mechanisms. Virulence 4:119–128. doi:10.4161/viru.22913. 23302789 PMC3654610

[24] Schlecht LM, Peters BM, Krom BP, Freiberg JA, Hänsch GM, Filler SG, Jabra-Rizk MA, Shirtliff ME 2015 Systemic *Staphylococcus aureus* infection mediated by *Candida albicans* hyphal invasion of mucosal tissue. Microbiology 161:168–181. doi:10.1099/mic.0.083485-0. 25332378 PMC4274785

[25] El-Azizi MA, Starks SE, Khardori N 2004 Interactions of *Candida albicans* with other *Candida* spp. and bacteria in the biofilms. J Appl Microbiol 96:1067–1073. doi:10.1111/j.1365-2672.2004.02213.x. 15078523

[26] Fox EP, Cowley ES, Nobile CJ, Hartooni N, Newman DK, Johnson AD 2014 Anaerobic bacteria grow within *Candida albicans* biofilms and induce biofilm formation in suspension cultures. Curr Biol 24:2411–2416. doi:10.1016/j.cub.2014.08.057. 25308076 PMC4252622

[27] Harriott MM, Noverr MC 2010 Ability of *Candida albicans* mutants to induce *Staphylococcus aureus* vancomycin resistance during polymicrobial biofilm formation. Antimicrob Agents Chemother 54:3746–3755. doi:10.1128/AAC.00573-10. 20566760 PMC2934986

[28] Mukherjee PK, Sendid B, Hoarau G, Colombel J-F, Poulain D, Ghannoum MA 2015 Mycobiota in gastrointestinal diseases. Nat Rev Gastroenterol Hepatol 12:77–87. doi:10.1038/nrgastro.2014.188. 25385227

[29] Mason KL, Erb Downward JR, Mason KD, Falkowski NR, Eaton KA, Kao JY, Young VB, Huffnagle GB 2012 *Candida albicans* and bacterial microbiota interactions in the cecum during recolonization following broad-spectrum antibiotic therapy. Infect Immun 80:3371–3380. doi:10.1128/IAI.00449-12. 22778094 PMC3457555

[30] Raponi G, Visconti V, Brunetti G, Ghezzi MC 2014 *Clostridium difficile* infection and *Candida* colonization of the gut: is there a correlation? Clin Infect Dis 59:1648–1649. doi:10.1093/cid/ciu637. 25091308

[31] Guastalegname M, Russo A, Falcone M, Giuliano S, Venditti M 2013 Candidemia subsequent to severe infection due to *Clostridium difficile*: is there a link? Clin Infect Dis 57:772–774. doi:10.1093/cid/cit362. 23723197

[32] Russo A, Falcone M, Fantoni M, Murri R, Masucci L, Carfagna P, Ghezzi MC, Posteraro B, Sanguinetti M, Venditti M 2015 Risk factors and clinical outcomes of candidaemia in patients treated for *Clostridium difficile* infection. Clin Microbiol Infect 21:493.e1–493.e4. doi:10.1016/j.cmi.2014.12.024. 25698658

[33] Manian FA, Bryant A 2013 Does *Candida* species overgrowth protect against *Clostridium difficile* infection? Clin Infect Dis 56:464–465. doi:10.1093/cid/cis854. 23042967

[34] Nerandzic MM, Mullane K, Miller MA, Babakhani F, Donskey CJ 2012 Reduced acquisition and overgrowth of vancomycin-resistant enterococci and *Candida* species in patients treated with fidaxomicin versus vancomycin for *Clostridium difficile* infection. Clin Infect Dis 55(Suppl 2):S121–S126. doi:10.1093/cid/cis440. 22752860 PMC3388028

[35] Dumitru R, Hornby JM, Nickerson KW 2004 Defined anaerobic growth medium for studying *Candida albicans* basic biology and resistance to eight antifungal drugs. Antimicrob Agents Chemother 48:2350–2354. doi:10.1128/AAC.48.7.2350-2354.2004. 15215080 PMC434226

[36] Webster CE, Odds FC 1987 Growth of pathogenic *Candida* isolates anaerobically and under elevated concentrations of CO_2_ in air. J Med Vet Mycol 25:47–53. doi:10.1080/02681218780000061. 3106612

[37] Hall IC, O’Toole E 1935 Intestinal flora in new-born infants: with a description of a new pathogenic anaerobe, *Bacillus difficilis*. Am J Dis Child 49:390–402. doi:10.1001/archpedi.1935.01970020105010.

[38] He G, Shankar RA, Chzhan M, Samouilov A, Kuppusamy P, Zweier JL 1999 Noninvasive measurement of anatomic structure and intraluminal oxygenation in the gastrointestinal tract of living mice with spatial and spectral EPR imaging. Proc Natl Acad Sci U S A 96:4586–4591. doi:10.1073/pnas.96.8.4586. 10200306 PMC16376

[39] Wang LH, He Y, Gao Y, Wu JE, Dong YH, He C, Wang SX, Weng LX, Xu JL, Tay L, Fang RX, Zhang LH 2004 A bacterial cell-cell communication signal with cross-kingdom structural analogues. Mol Microbiol 51:903–912. doi:10.1046/j.1365-2958.2003.03883.x. 14731288

[40] Boon C, Deng Y, Wang L-H, He Y, Xu J-L, Fan Y, Pan SQ, Zhang L-H 2008 A novel DSF-like signal from *Burkholderia cenocepacia* interferes with *Candida albicans* morphological transition. ISME J 2:27–36. doi:10.1038/ismej.2007.76. 18049456

[41] McLean RJC 2014 Normal bacterial flora may inhibit *Candida albicans* biofilm formation by autoinducer-2. Front Cell Infect Microbiol 4:117. doi:10.3389/fcimb.2014.00117. 25221750 PMC4147847

[42] Shareck J, Belhumeur P 2011 Modulation of morphogenesis in *Candida albicans* by various small molecules. Eukaryot Cell 10:1004–1012. doi:10.1128/EC.05030-11. 21642508 PMC3165445

[43] Levett PN 1987 Production of p-cresol by *Clostridium difficile* on different basal media. Lett Appl Microbiol 5:71–73. doi:10.1111/j.1472-765X.1987.tb01617.x.

[44] Sivsammye G, Sims HV 1990 Presumptive identification of *Clostridium difficile* by detection of p-cresol in prepared peptone yeast glucose broth supplemented with p-hydroxyphenylacetic acid. J Clin Microbiol 28:1851–1853. 2394806 10.1128/jcm.28.8.1851-1853.1990 PMC268058

[45] Hafiz S, Oakley CL 1976 *Clostridium difficile*: isolation and characteristics. J Med Microbiol 9:129–136. doi:10.1099/00222615-9-2-129. 933146

[46] Nowak A, Libudzisz Z 2006 Influence of phenol, p-cresol and indole on growth and survival of intestinal lactic acid bacteria. Anaerobe 12:80–84. doi:10.1016/j.anaerobe.2005.10.003. 16701619

[47] Heipieper HJ, Keweloh H, Rehm HJ 1991 Influence of phenols on growth and membrane permeability of free and immobilized *Escherichia coli*. Appl Environ Microbiol 57:1213–1217. 2059043 10.1128/aem.57.4.1213-1217.1991 PMC182870

[48] Selmer T, Andrei PI 2001 p-Hydroxyphenylacetate decarboxylase from *Clostridium difficile*. Eur J Biochem 268:1363–1372. doi:10.1046/j.1432-1327.2001.02001.x. 11231288

[49] Andrei PI, Pierik AJ, Zauner S, Andrei-Selmer LC, Selmer T 2004 Subunit composition of the glycyl radical enzyme p-hydroxyphenylacetate decarboxylase. A small subunit, HpdC, is essential for catalytic activity. Eur J Biochem 271:2225–2230. doi:10.1111/j.1432-1033.2004.04152.x. 15153112

[50] Dawson LF, Donahue EH, Cartman ST, Barton RH, Bundy J, McNerney R, Minton NP, Wren BW 2011 The analysis of para-cresol production and tolerance in *Clostridium difficile* 027 and 012 strains. BMC Microbiol 11:86. doi:10.1186/1471-2180-11-86. 21527013 PMC3102038

[51] Cartman ST, Kelly ML, Heeg D, Heap JT, Minton NP 2012 Precise manipulation of the *Clostridium difficile* chromosome reveals a lack of association between the *tcdC* genotype and toxin production. Appl Environ Microbiol 78:4683–4690. doi:10.1128/AEM.00249-12. 22522680 PMC3370502

[52] Bachtiar EW, Bachtiar BM, Jarosz LM, Amir LR, Sunarto H, Ganin H, Meijler MM, Krom BP 2014 AI-2 of *Aggregatibacter actinomycetemcomitans* inhibits *Candida albicans* biofilm formation. Front Cell Infect Microbiol 4:94. doi:10.3389/fcimb.2014.00094. 25101248 PMC4104835

[53] Sproule-Willoughby KM, Stanton MM, Rioux KP, McKay DM, Buret AG, Ceri H 2010 *In vitro* anaerobic biofilms of human colonic microbiota. J Microbiol Methods 83:296–301. doi:10.1016/j.mimet.2010.09.020. 20920538

[54] Wezensky SJ, Cramer RA 2011 Implications of hypoxic microenvironments during invasive aspergillosis. Med Mycol 49(Suppl 1):S120–S124. doi:10.3109/13693786.2010.495139. 20560863 PMC2951492

[55] Land GA, McDonald WC, Stjernholm RL, Friedman L 1975 Factors affecting filamentation in *Candida albicans*: changes in respiratory activity of *Candida albicans* during filamentation. Infect Immun 12:119–127. 1095490 10.1128/iai.12.1.119-127.1975 PMC415254

[56] Somerville GA, Proctor RA 2013 Cultivation conditions and the diffusion of oxygen into culture media: the rationale for the flask-to-medium ratio in microbiology. BMC Microbiol 13:9. doi:10.1186/1471-2180-13-9. 23324109 PMC3551634

[57] Dione N, Khelaifia S, Lagier J-C, Raoult D 2015 The aerobic activity of metronidazole against anaerobic bacteria. Int J Antimicrob Agents 45:537–540. doi:10.1016/j.ijantimicag.2014.12.032. 25813393

[58] Marteyn B, Scorza FB, Sansonetti PJ, Tang C 2011 Breathing life into pathogens: the influence of oxygen on bacterial virulence and host responses in the gastrointestinal tract. Cell Microbiol 13:171–176. doi:10.1111/j.1462-5822.2010.01549.x. 21166974

[59] Rigottier-Gois L 2013 Dysbiosis in inflammatory bowel diseases: the oxygen hypothesis. ISME J 7:1256–1261. doi:10.1038/ismej.2013.80. 23677008 PMC3695303

[60] Theriot CM, Koenigsknecht MJ, Carlson PE, Hatton GE, Nelson AM, Li B, Huffnagle GB, Li J, Young VB 2014 Antibiotic-induced shifts in the mouse gut microbiome and metabolome increase susceptibility to *Clostridium difficile* infection. Nat Commun 5:3114. doi:10.1038/ncomms4114. 24445449 PMC3950275

[61] Hansen AK, Nielsen DS 2014 Handbook of laboratory animal bacteriology, 2nd ed, p 133–166. CRC Press, Boca Raton, FL.

[62] Krom BP, Levy N, Meijler MM, Jabra-Rizk MA 2016 Farnesol and *Candida albicans*: quorum sensing or not quorum sensing? Isr J Chem 56:295–301. doi:10.1002/ijch.201500025.

[63] Ha KC, White TC 1999 Effects of azole antifungal drugs on the transition from yeast cells to hyphae in susceptible and resistant isolates of the pathogenic yeast *Candida albicans*. Antimicrob Agents Chemother 43:763–768. 10103178 10.1128/aac.43.4.763 PMC89204

[64] Brand A 2012 Hyphal growth in human fungal pathogens and its role in virulence. Int J Microbiol 2012:517529. doi:10.1155/2012/517529. 22121367 PMC3216317

[65] Low CF, Chong PP, Yong PV, Lim CS, Ahmad Z, Othman F 2008 Inhibition of hyphae formation and SIR2 expression in *Candida albicans* treated with fresh *Allium sativum* (garlic) extract. J Appl Microbiol 105:2169–2177. doi:10.1111/j.1365-2672.2008.03912.x. 19120662

[66] Khan A, Ahmad A, Ahmad Khan L, Padoa CJ, van Vuuren S, Manzoor N 2015 Effect of two monoterpene phenols on antioxidant defense system in *Candida albicans*. Microb Pathog 80:50–56. doi:10.1016/j.micpath.2015.02.004. 25681060

[67] Cordeiro RDA, Teixeira CE, Brilhante RS, Castelo-Branco DS, Alencar LP, de Oliveira JS, Monteiro AJ, Bandeira TJ, Sidrim JJ, Moreira JL, Rocha MF 2015 Exogenous tyrosol inhibits planktonic cells and biofilms of Candida species and enhances their susceptibility to antifungals. FEMS Yeast Res 15:fov012. doi:10.1093/femsyr/fov012. 25795651

[68] Monteiro DR, Feresin LP, Arias LS, Barão VA, Barbosa DB, Delbem AC 2015 Effect of tyrosol on adhesion of *Candida albicans* and *Candida glabrata* to acrylic surfaces. Med Mycol 53:656–665. doi:10.1093/mmy/myv052. 26162470

[69] Freires IA, Queiroz VC, Furletti VF, Ikegaki M, de Alencar SM, Duarte MC, Rosalen PL 2016 Chemical composition and antifungal potential of Brazilian propolis against *Candida* spp. J Mycol Med 26:122–132. doi:10.1016/j.mycmed.2016.01.003. 26916845

[70] Panwar R, Pemmaraju SC, Sharma AK, Pruthi V 2016 Efficacy of ferulic acid encapsulated chitosan nanoparticles against *Candida albicans* biofilm. Microb Pathog 95:21–31. doi:10.1016/j.micpath.2016.02.007. 26930164

[71] De Vita D, Friggeri L, D’Auria FD, Pandolfi F, Piccoli F, Panella S, Palamara AT, Simonetti G, Scipione L, Di Santo R, Costi R, Tortorella S 2014 Activity of caffeic acid derivatives against *Candida albicans* biofilm. Bioorg Med Chem Lett 24:1502–1505. doi:10.1016/j.bmcl.2014.02.005. 24582984

[72] Pointer BR, Boyer MP, Schmidt M 2015 Boric acid destabilizes the hyphal cytoskeleton and inhibits invasive growth of *Candida albicans*. Yeast 32:389–398. doi:10.1002/yea.3066. 25612315

[73] Doke SK, Raut JS, Dhawale S, Karuppayil SM 2014 Sensitization of *Candida albicans* biofilms to fluconazole by terpenoids of plant origin. J Gen Appl Microbiol 60:163–168. doi:10.2323/jgam.60.163. 25420420

[74] Fazly A, Jain C, Dehner AC, Issi L, Lilly EA, Ali A, Cao H, Fidel PL, Rao RP, Kaufman PD 2013 Chemical screening identifies filastatin, a small molecule inhibitor of *Candida albicans* adhesion, morphogenesis, and pathogenesis. Proc Natl Acad Sci U S A 110:13594–13599. doi:10.1073/pnas.1305982110. 23904484 PMC3746938

[75] Pierce CG, Chaturvedi AK, Lazzell AL, Powell AT, Saville SP, McHardy SF, Lopez-Ribot JL 2015 A novel small molecule inhibitor of *Candida albicans* biofilm formation, filamentation and virulence with low potential for the development of resistance. NPJ Biofilms Microbiomes 1:15012. doi:10.1038/npjbiofilms.2015.12. 26691764 PMC4681527

[76] Van Eijk E, Anvar SY, Browne HP, Leung WY, Frank J, Schmitz AM, Roberts AP, Smits WK 2015 Complete genome sequence of the *Clostridium difficile* laboratory strain 630Δerm reveals differences from strain 630, including translocation of the mobile element CTn5. BMC Genomics 16:31. doi:10.1186/s12864-015-1252-7. 25636331 PMC4320837

[77] Hussain HA, Roberts AP, Mullany P 2005 Generation of an erythromycin-sensitive derivative of *Clostridium difficile* strain 630 (630Deltaerm) and demonstration that the conjugative transposon Tn916DeltaE enters the genome of this strain at multiple sites. J Med Microbiol 54:137–141. doi:10.1099/jmm.0.45790-0. 15673506

[78] Myers GS, Rasko DA, Cheung JK, Ravel J, Seshadri R, DeBoy RT, Ren Q, Varga J, Awad MM, Brinkac LM, Daugherty SC, Haft DH, Dodson RJ, Madupu R, Nelson WC, Rosovitz MJ, Sullivan SA, Khouri H, Dimitrov GI, Watkins KL, Mulligan S, Benton J, Radune D, Fisher DJ, Atkins HS, Hiscox T, Jost BH, Billington SJ, Songer JG, McClane BA, Titball RW, Rood JI, Melville SB, Paulsen IT 2006 Skewed genomic variability in strains of the toxigenic bacterial pathogen, *Clostridium perfringens*. Genome Res 16:1031–1040. doi:10.1101/gr.5238106. 16825665 PMC1524862

[79] Fonzi WA, Irwin MY 1993 Isogenic strain construction and gene mapping in *Candida albicans*. Genetics 134:717–728. 8349105 10.1093/genetics/134.3.717 PMC1205510

[80] Gibson DG, Young L, Chuang R-Y, Venter JC, Hutchison CA, Smith HO 2009 Enzymatic assembly of DNA molecules up to several hundred kilobases. Nat Methods 6:343–345. doi:10.1038/nmeth.1318. 19363495

[81] Purdy D, O’Keeffe TA, Elmore M, Herbert M, McLeod A, Bokori-Brown M, Ostrowski A, Minton NP 2002 Conjugative transfer of clostridial shuttle vectors from *Escherichia coli* to *Clostridium difficile* through circumvention of the restriction barrier. Mol Microbiol 46:439–452. doi:10.1046/j.1365-2958.2002.03134.x. 12406220

[82] Benoit MR, Conant CG, Ionescu-Zanetti C, Schwartz M, Matin A 2010 New device for high-throughput viability screening of flow biofilms. Appl Environ Microbiol 76:4136–4142. doi:10.1128/AEM.03065-09. 20435763 PMC2897429

[83] Schneider CA, Rasband WS, Eliceiri KW 2012 NIH Image to ImageJ: 25 years of image analysis. Nat Methods 9:671–675. doi:10.1038/nmeth.2089. 22930834 PMC5554542

[84] Krom BP, Cohen JB, McElhaney Feser GE, Cihlar RL 2007 Optimized candidal biofilm microtiter assay. J Microbiol Methods 68:421–423. doi:10.1016/j.mimet.2006.08.003. 17005276

[85] Kirk JA, Fagan RP 2016 Heat shock increases conjugation efficiency in *Clostridium difficile*. Anaerobe 42:1–5. doi:10.1016/j.anaerobe.2016.06.009. 27377776 PMC5154368

