# Supplementary figures and images for: Interspecies Interactions between Clostridium difficile and Candida albicans
Source: mSphere. 2016 Nov 9;1(6):e00187-16. doi: 10.1128/mSphere.00187-16 (PMC5103046; doi:10.1128/mSphere.00187-16)

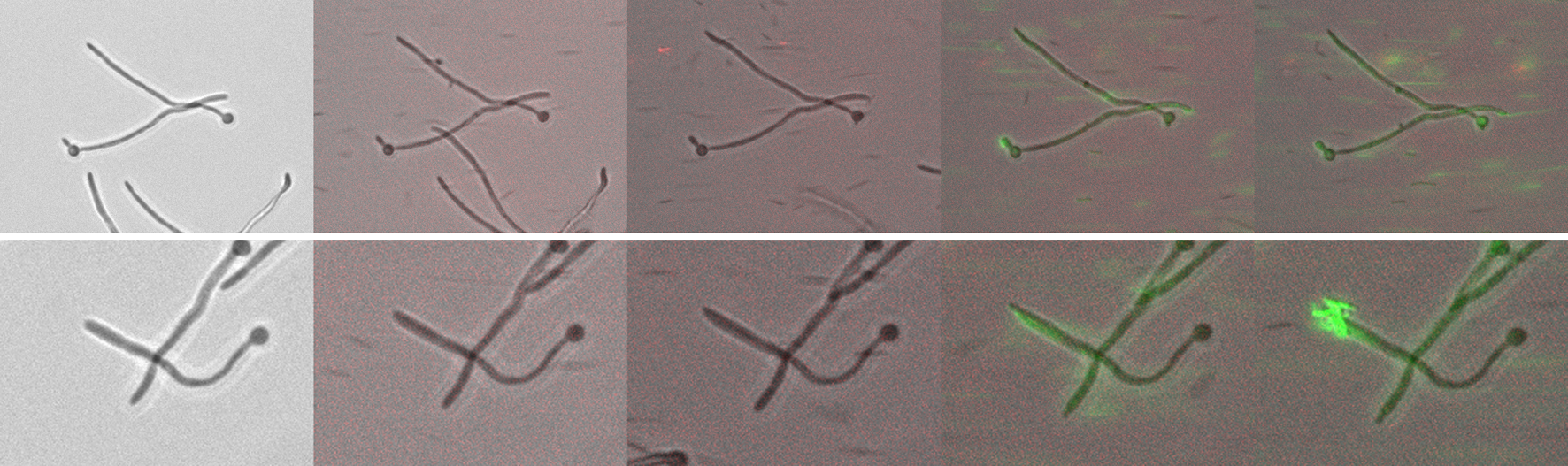

Supplement: Figure S1 [file sph005162174sf1.tif]
